# Clonal Spread and Genetic Mechanisms Underpinning Ciprofloxacin Resistance in *Salmonella enteritidis*

**DOI:** 10.3390/foods14020289

**Published:** 2025-01-16

**Authors:** Zengfeng Zhang, Hang Zhao, Chunlei Shi

**Affiliations:** MOST-USDA Joint Research Center for Food Safety, School of Agriculture and Biology and State Key Laboratory of Microbial Metabolism, Shanghai Jiao Tong University, Shanghai 200240, China; zzfffjp241@sjtu.edu.cn (Z.Z.); zh19940516@126.com (H.Z.)

**Keywords:** *Salmonella enteritidis*, ciprofloxacin resistance, PMQR, *gyr*A, efflux pump

## Abstract

*Salmonella enteritidis* is a major cause of foodborne illness worldwide, and the emergence of ciprofloxacin-resistant strains poses a significant threat to food safety and public health. This study aimed to investigate the prevalence, spread, and mechanisms of ciprofloxacin resistance in *S. enteritidis* isolates from food and patient samples in Shanghai, China. A total of 1625 *S. enteritidis* isolates were screened, and 34 (2.1%) exhibited resistance to ciprofloxacin. Pulsed-field gel electrophoresis (PFGE) results suggested that clonal spread might have persisted among these 34 isolates in the local area for several years. Multiple plasmid-mediated quinolone resistance (PMQR) genes, GyrA mutations in the quinolone resistance-determining region (QRDR), and overexpression of RND efflux pumps were identified as potential contributors to ciprofloxacin resistance. PMQR genes *oqx*AB, *qnr*A, *qnr*B, and *aac*(6’)-Ib-cr as well as GyrA mutations S83Y, S83R, D87Y, D87G, D87N, and S83Y-D87Y were identified. The co-transfer of the PMQR gene *oqx*AB with the ESBL gene *bla*_CTX-M-14/55_ on an IncHI2 plasmid with a size of ~245 kbp was observed through conjugation, highlighting the role of horizontal gene transfer in the dissemination of antibiotic resistance. Sequencing of the *oqx*AB-bearing plasmid p12519A revealed a 248,746 bp sequence with a typical IncHI2 backbone. A 53,104 bp multidrug resistance region (MRR) was identified, containing two key antibiotic resistance determinants: IS*26*-*oqx*R-*oqx*AB-IS*26* and IS*26*-ΔIS*Ecp1*-*bla*_CTX-M-14_-IS*903B*. The findings of this study indicate that ciprofloxacin-resistant *S. Enteritidis* poses a significant threat to food safety and public health. The persistence of clonal spread and the horizontal transfer of resistance genes highlight the need for enhanced surveillance and control measures to prevent the further spread of antibiotic resistance.

## 1. Introduction

*Salmonella* is one of the foremost foodborne pathogens affecting both humans and animals globally. According to estimates by the United States Centers for Disease Control and Prevention (USCDC), approximately 1.35 million infections, 26,500 hospitalizations, and 420 deaths are caused by *Salmonella* in the United States annually (https://www.cdc.gov/Salmonella/ available by 12 November 2024). Among 2659 serotypes, *S. enteritidis* is the most common serovar that caused human salmonellosis in China, USA, and EU [1,2,3,4,5]. In China, *S. enteritidis* was the serovar most frequently found in poultry meat, accounting for from 25.2% to 45.7% [6,7,8,9]. Further, this pathogen can be transferred to humans via contact with contaminated poultry meat and egg products [10]. Fluoroquinolones are the preferred drug of choice for treating human infections caused by *Salmonella* [11,12,13]. However, in recent years, ciprofloxacin resistance has emerged in *S. Enteritidis*, a representative drug of the fluoroquinolone class [14,15,16]. Resistance to ciprofloxacin in *S. Enteritidis* isolates from foods and patients has been reported to range from 3.8% to 8.3% [14,15,16]. The emergence of fluoroquinolone resistance in *S. enteritidis* requires the alteration of treatment strategies and poses a serious threat to food safety and public health.

Mutations in the quinolone resistance-determining region (QRDR) of the DNA gyrase subunits A (GyrA) and B (GyrB), as well as the topoisomerase IV subunits C (ParC) and E (ParE), have been confirmed to result in reduced susceptibility to fluoroquinolones [17]. Simultaneous mutations in GyrA and ParC were frequently identified in *S. enteritidis* and *S.* Indiana isolates with resistance to ciprofloxacin [18,19,20]. Currently, only mutations in GyrA were found in *S. enteritidis* , while GyrB, ParC, and ParE mutations were absent [2,21]. Plasmid-mediated quinolone resistance (PMQR) genes and the overexpression of efflux pumps could also contribute to low-level resistance to fluoroquinolones [17,18,22]. The high-level expression of the Resistance-Nodulation-Division (RND) superfamily have been shown to result in increased fluoroquinolone resistance through exporting substrates out of cells in Gram-negative bacteria [17]. PMQR genes are generally carried by conjugative plasmids, which facilitate their dissemination among *Salmonella* and other bacteria [14,23,24,25]. Four types of PMQR genes were discovered, including *qnr*, *aac*(6’)-Ib-cr, *oqx*AB, and *qep*A [17]. In recent years, PMQR genes have become more prevalent, but mutations in QRDR have decreased in fluoroquinolone-resistant *Salmonella* [24,26,27,28]. Moreover, conjugative plasmids carrying multiple PMQR genes such as *aac*(6’)-Ib-cr and *qnr*B could mediate above the breakpoint level of resistance to ciprofloxacin, and this mechanism has been found in *S.* Agona, *S.* Derby, *S.* Thompsom, and *S.* London [26,27,29]. Hence, PMQR genes can be considered as having an important role in the recent increasing ciprofloxacin resistance in *Salmonella* isolates.

In this study, the prevalence of ciprofloxacin resistance in *S. enteritidis* isolates on a large scale was investigated. The investigation involved the collection of *S. Enteritidis* isolates from various food samples as well as from clinical specimens from patients with salmonellosis. Ciprofloxacin resistance determinants, including QRDR mutations, PMQR genes, and efflux pumps, were characterized. Additionally, the clonal relationship of isolates and transferability of plasmids were also examined.

## 2. Materials and Methods

### 2.1. Bacterial Isolates and Antimicrobial Susceptibility Testing

A total of 1625 *S. enteritidis* isolates were recovered from patients and retail foods in Shanghai, China, from 2011 to 2013. The food sources encompassed a wide range, including chicken, pork, duck, marine products, and frozen foods. As for human sources, isolates were obtained from the stools and blood samples of outpatients and inpatients seeking treatment for diarrhea in hospitals. *Salmonella* isolates were identified and serotyped by utilizing commercial antiserum (Statens Serum Institute, Copenhagen, Denmark) and API20E test strips (BioMerieux, Marcy-l'Étoile , France), both employed in accordance with the manufacturers’ instructions.

Ciprofloxacin-resistant *Salmonella* isolates were selected on Mueller–Hinton agar plates containing 1 µg/mL ciprofloxacin. The agar dilution method as recommended by the Clinical and Laboratory Standard Institute was used to identify Minimum Inhibitory Concentrations (MICs) of antibiotics to isolates [30]. The antibiotics tested were the following: amikacin (AMK), ampicillin (AMP), ceftiofur (TIO), ceftriaxone (CRO), cefoxitin (FOX), ceftazidime (CAZ), cefotaxime (CTX), nalidixic acid (NAL), chloramphenicol (CHL), kanamycin (KAN), gentamicin (GEN), streptomycin (STR), tetracycline (TET), sulfisoxazole (FIS), sulfamethoxazole/trimethoprim (SXT), azithromycin (AZM), meropenem (MEM), and imipenem (IMP). Broth microdilution as recommended by the European Committee was used to identify the susceptibility of colistin (CT) [31]. All antibiotics were purchased from Sigma-Aldrich Shanghai Trading Co. Ltd., Shanghai, China. *Escherichia coli* ATCC 25922 and *Enterococcus faecalis* ATCC 29212 were used as quality control strains in the MIC determination.

### 2.2. PCR and DNA Sequencing of Quinolone and Β-Lactamase Resistance Determination Genes

The QRDR genes of *gyr*A, *gyr*B, *par*C, and *par*E and PMQR genes of *qnr*A, *qnr*B, *qnr*S, *oqx*AB, *qep*A, and *aac(6’)-Ib* were amplified by PCR. 

Extended-Spectrum Beta-Lactamase (ESBL) genes *bla*_CTX-M_, *bla*_PSE_, *bla*_PER_, *bla*_OXA_, *bla*_CMY_, and *bla*_TEM_ as well as carbapenemase genes *bla*_IMP_, *bla*_VIM_, *bla*_KPC_, *bla*_SME_, *bla*_IMI_, *bla*_GES_, and *bla*_VEB_ were identified as described previously [32]. Multiplex PCR assays were further performed to identify *bla*_CTX-M_ subtypes as described previously [33].

For *gyr*A, *gyr*B, *par*C, *par*E, and *β*-lactamase gene sequences analysis, the PCR products were purified utilizing the TaKaRa Agarose Gel DNA Purification Kit (Version 2.0; TaKaRa). Subsequently, these purified products were sent to Shanghai Sunny Biotechnology Co., Ltd. (Shanghai, China), for sequencing. The obtained DNA sequence data were then analyzed and aligned using the BLAST tool (http://www.ncbi.nlm.nih.gov/BLAST/, accessed on 1 October 2024).

### 2.3. Pulsed-Field Gel Electrophoresis (PFGE)

PFGE was conducted to determine the genotypic relationship of the *S. enteritidis* isolates with *Xba* I digestion as previously described [34]. Briefly, the process began with the immobilization of *Salmonella* cells within SeaKem Gold agarose (Cambrex Bio Science, Walkersville, MD, USA). Following this, the cells were lysed to release their DNA, which was then embedded within the agarose matrix. The embedded DNA underwent digestion with 50 units of *Xba* I enzymes (sourced from Takara Biotechnology, Dalian, China) in a 37 °C water bath for a duration of 1.5 to 2 h. The restricted DNA fragments were then separated by electrophoresis in 0.5× Tris-borate-EDTA buffer at 14 °C for a period of 19 h, using a Chef-Mapper electrophoresis system (Bio-Rad, Richmond, CA, USA). The electrophoresis was conducted with pulse times ranging from 2.16 to 63.8 s, which allowed for the effective separation of the DNA fragments based on their size. Finally, the PFGE images were analyzed using BioNumerics Software 7.6 (Applied-Maths, Kortrijk, Belgium) to determine the *Salmonella* genotypes and their relatedness. *S.* Braenderup H9812 was used as the DNA size marker.

### 2.4. Conjugation Experiment and Plasmid Analysis

Conjugation experiments were conducted according to established protocols, utilizing *E. coli* C600 as the recipient strain as previously reported [35]. In these experiments, *Salmonella* served as the donor strain and was co-incubated with the recipient for an overnight period. Following incubation, the mixture was plated onto filter paper placed on an LB agar plate and allowed to culture for another overnight. To select for transconjugants, MacConkey agar plates containing ceftriaxone (4 µg/mL) and rifampin (200 µg/mL) were employed. PCR-based replicon typing, utilizing a set of 18 replicon primers, was performed on the transconjugants as described previously [36], and PFGE with S1 nuclease (Takara Biotechnology, Dalian, China) digestion was carried out to determine the size of the plasmid. A phage Lambda PFGE ladder (New England BioLabs, Ipswich, MA, USA) was used as the DNA size marker.

### 2.5. Efflux Pump Inhibitor Test by Using Phe-Arg-β-Naphthylamide (PAβN)

To determine whether the overexpression of the efflux pump affected quinolone resistance, ciprofloxacin susceptibility was compared by the broth dilution method in the presence (100 μg/mL) or absence of PAβN (Sigma-Aldrich, Munich, Germany). The concentration of PAβN applied was 0.25-fold MIC of ciprofloxacin to the isolates (ranging from 0.015 to 2 μg/mL); under this concentration, an inhibitory effect of the inhibitor itself could be eliminated [37].

### 2.6. Whole-Genome Sequencing of S. Enteritidis SJTUF12519

Overnight cultures of *Salmonella enteritidis* strain SJTUF12519 cells were meticulously collected to proceed with the extraction of genomic DNA. This was achieved using the QIAamp DNA Mini Kit (Qiagen, Redwood City, CA, USA), strictly following the manufacturer’s detailed instructions to ensure high-quality DNA extraction. For comprehensive genomic analysis, whole-genome sequencing (WGS) was entrusted to Shanghai Personal Biotechnology Co., Ltd. (Shanghai, China), utilizing two advanced sequencing platforms: the PacBio RS II system (Pacific Biosciences, Menlo Park, CA, USA) and the Illumina MiSeq (Illumina, San Diego, CA, USA).

For the PacBio RS II platform, a 10 kilobase pair (kbp) DNA library was meticulously constructed, followed by sequencing using single-molecule real-time (SMRT) technology, which captures an entire genome with unparalleled accuracy. The sequence data obtained from the PacBio RS II platform were subsequently assembled using Canu software 1.8 [38]. In parallel, for the Illumina MiSeq platform, a 400 base pair (bp) DNA library was prepared and sequenced in paired-end mode, generating complementary sequence data. The SPAdes assembler [39] was employed to piece together the Illumina MiSeq data, enhancing the completeness and accuracy of the genome assembly.

Upon integrating the data from both sequencing platforms, the consensus genome sequence was determined using Pilon software [40]. Annotation of the genome was performed using a suite of bioinformatics tools, including RAST (http://rast.nmpdr.org, accessed on 1 October 2024), BLASTn, and BLASTp (http://blast.ncbi.nlm.nih.gov/Blast.cgi, accessed on 1 October 2024), which provided insights into the functional genes and proteins within the genome. Additionally, the ORF Finder tool (http://www.ncbi.nlm.nih.gov/orffinder, accessed on 1 October 2024) was used to identify open reading frames (ORFs), revealing potential gene expression and protein synthesis regions.

To further investigate the plasmid content of the *Salmonella* strain, the oriTfinder tool (https://bioinfo-mml.sjtu.edu.cn/oriTfinder/, accessed on 1 October 2024) was employed to identify the origin of transfers in bacterial plasmid DNA sequences. The plasmid type was accurately identified using Plasmidfinder (https://cge.food.dtu.dk/services/PlasmidFinder/, accessed on 1 October 2024), facilitating the classification and characterization of plasmids based on their sequence features.

### 2.7. Data Availability

The entire nucleotide sequences of p12519A are archived in the NCBI database, assigned with Accession Number CP041174.

## 3. Results and Discussion

### 3.1. Antimicrobial Susceptibility Test Results

**Figure 1:**
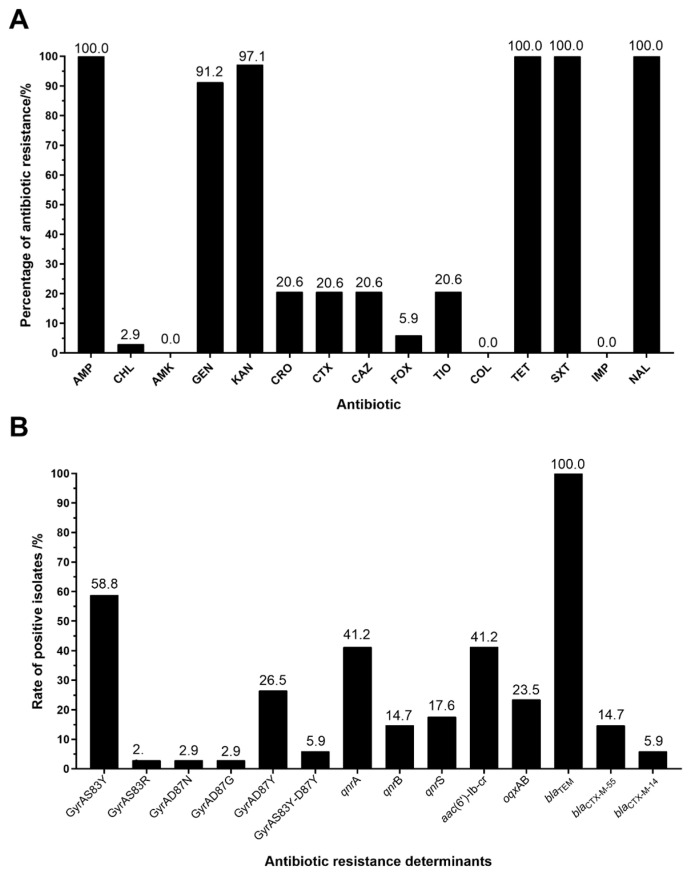
Prevalence of antibiotic resistance (**A**) and antibiotic resistance determinants (**B**) in ciprofloxacin-resistant *S. enteritidis* .

Among 1625 *S. enteritidis* isolates, 34 (2.1%) isolates were resistant to ciprofloxacin, which was lower than that (3.18%) in a previous study [2]. All these isolates were recovered from patient samples (Supplementary Materials). More importantly, 7 of 34 isolates exhibited resistance to ceftriaxone, ceftiofur, ceftazidime, and cefotaxime, and two isolates were resistant to cefoxitin. All of these 34 isolates were also resistant to ampicillin, tetracycline, trimethoprim-sulfamethoxazole, and nalidixic acid, and most of them were resistant to kanamycin (97.1%; 33/34), gentamicin (91.2%; 31/34) and chloramphenicol (2.9%; 1/34) (Figure 1A). These 34 isolates were susceptible to amikacin, colistin, meropenem, and imipenem. There were five different resistant patterns among these 34 *S. enteritidis* isolates, of which, AMP-KAN-GEN-TET-SXT-NAL-CIP (*n* = 24) was the predominant phenotype, and then AMP-KAN-GEN-TET-CRO-TIO-CAZ-CTX-SXT-NAL-CIP (*n* = 5).

The presence of MDR *S. Enteritidis* isolates, particularly those with resistance to both fluoroquinolones and third-generation cephalosporins in this study, was a significant food safety concern. Fluoroquinolones are often used as a first-line treatment for severe *Salmonella* infections [11,12], and the emergence of resistance to these antibiotics can lead to treatment failures and increased morbidity and mortality. Their co-resistance to cephalosporins further complicates treatment options, as these drugs are also important for treating severe infections. The high prevalence of resistance to multiple antibiotics, including ampicillin, tetracycline, and trimethoprim-sulfamethoxazole, suggested that these isolates may have acquired multiple resistance mechanisms, possibly through horizontal gene transfer. This finding underscores the urgent need for continuous monitoring and effective antibiotic stewardship programs to curb the spread of such resistant strains.

### 3.2. Prevalence and Distribution of Antibiotic Resistance Determinant

These 34 ciprofloxacin-resistant isolates were tested for mutations in the QRDR of *gyr*A and *par*C and PMQR genes of *qnr*A, *qnr*B, *qnr*S, *oqx*AB, *qep*A, and *aac*(6’)-Ib-cr. Amino acid substitutions were only found in GyrA, and mutations in GyrB, ParC, and ParE were absent. Mutations in GyrA were commonly identified as S83Y (58.8%; 20/34), D87Y (26.5%; 9/34), S83Y-D87Y(5.9%; 2/34), S83R (2.9%; 1/34), D87N (2.9%; 1/34), and D87G (2.9%; 1/34) (Figure 1B). *aac(6′)-Ib-cr* (41.2%), and *qnr*A (41.2%) of PMQR genes were most frequently identified, followed by *oqx*AB (23.5%), *qnr*S (17.6%), and *qnr*B (14.7%) (Figure 1B). No *qep*A was identified, which could be attributed to its low prevalence [2]. Of particular note, 33 of 34 isolates harbored at least one PMQR gene, and 10 isolates possessed at least two PMQR genes.

We also investigated ESBL genes in these 34 isolates by PCR and further sequences for subtyping. The ESBL gene *bla*_TEM-1_ (100.0%) was found in all 34 isolates. *bla*_CTX-M-55_ (*n* = 5) and *bla*_CTX-M-14_ (*n* = 2) were identified in seven isolates showing resistance to ceftriaxone. The combination of three PMQR genes *qnr*A-*oqx*AB*-aac(6′)-Ib-cr* was revealed in one *bla*_CTX-M-14_-positive isolate with amino acid substitution of S83Y in GyrA.

The high frequency of *gyr*A mutations in *S. Enteritidis*, particularly S83Y and D87Y, was consistent with previous studies [2] and indicated that these mutations are a major mechanism of ciprofloxacin resistance in *S. Enteritidis*. In this study, the presence of multiple PMQR genes, especially *aac(6′)-Ib-cr* and *qnr*A, suggested that these genes play a significant role in the development of low-level ciprofloxacin resistance. The co-occurrence of multiple PMQR genes in some isolates, along with *gyr*A mutations, likely contributes to higher levels of ciprofloxacin resistance. The presence of ESBL genes, such as *bla*_CTX-M-55_ and *bla*_CTX-M-14_, in these isolates further emphasizes the complexity of their resistance profile and the potential for these isolates to spread resistance to other bacteria.

### 3.3. PFGE Analysis

**Figure 2:**
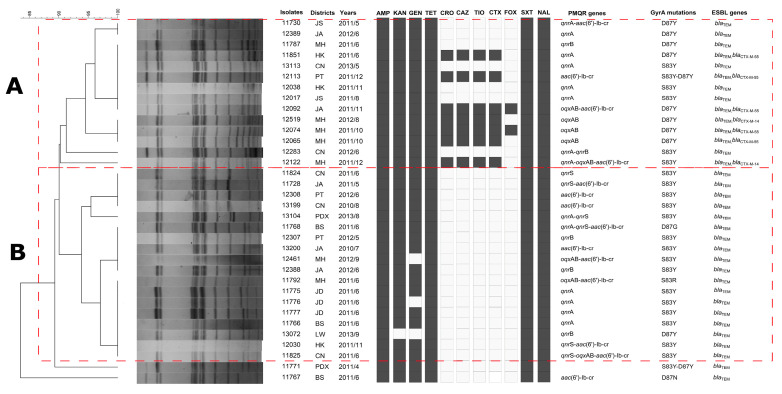
Pulsed-field gel electrophoresis (PFGE) *Xba* I patterns of *S. enteritidis* isolates with resistance to ciprofloxacin. Cluster analysis and band-matching applications within the BioNumerics Software 7.6 (Applied-Maths, Kortrijk, Belgium) were utilized to evaluate the similarity of PFGE patterns. BS, Baoshan District; JS, Jinshan District; JA, Jianan District; JD Jiading District; LW, Luwan District; MH, Minghang District; HK, Hongkou District; CN, Changning District; PT, Putuo district; PDX, Pudong District.

The clonal relationship of the 34 ciprofloxacin-resistant *S. enteritidis* isolates was studied by PFGE. A total of 11 genotypes were observed, and two clusters (A and B) were identified by a 90% similarity of PFGE patterns (Figure 2). The main difference between cluster A and B was cephalosporin resistance. All the cephalosporin-resistant isolates belonged to cluster A and shared one PFGE profile. For example, fluoroquinolone- and cephalosporin-co-resistant isolates 12519, 12074, and 12065 shared one profile with a similar antibiotic resistance phenotype, PMQR genes, ESBL genes, and mutations in GyrA, and they were recovered from the same districts from 2011 to 2012 (Figure 2). Moreover, isolates 11787, 11851, 13113, 12113, 12038, 12017, and 12092 collected from different districts in Shanghai from 2011 to 2013 were also grouped into the same cluster. Similar results were also found in cluster B (Figure 2), suggesting that there is clonal spread of these *S. enteritidis* isolates from different foods and patients in the locality.

The clustering of isolates with similar resistance profiles and genetic backgrounds suggests that there was clonal spread of ciprofloxacin-resistant *S. Enteritidis* in the region. The presence of a single PFGE profile among the cephalosporin-resistant isolates in cluster A, and the recovery of these isolates from the same districts over a period of time, indicated that these strains may have persisted in the environment and/or food supply. The clonal spread of these isolates highlighted the importance of monitoring and controlling the sources of contamination, such as poultry farms and food processing facilities, to prevent the further dissemination of these resistant strains.

### 3.4. Influence of Efflux Pump Inhibitors

The MICs of ciprofloxacin in the *Salmonella* isolates were tested in the presence of a 0.25-fold MIC of the efflux pump inhibitor PAβN (Table 1). Overexpression of the efflux pump was found to be common in ciprofloxacin-resistant *S. enteritidis* isolates. The MICs of ciprofloxacin in all the isolates decreased by at least eight-fold after adding PAβN. The median MIC value of the ciprofloxacin-resistant isolates was 1 μg/mL (1–8 μg/mL), and this value dropped to 0.125 μg/mL (0.06–0.5 μg/mL) when PAβN was added. After PAβN was added, the ciprofloxacin-resistant isolates (*n* = 7) become ciprofloxacin-susceptible isolates (*n* = 6) or ciprofloxacin-intermediate isolates (*n* = 1) based on CLSI 2019 breakpoints. 

The decrease in the MICs of ciprofloxacin may have resulted from inhibition of the RND efflux pump AcrAB-TolC, which was able to export fluoroquinolones [40,41]. The varied expression of the AcrAB-TolC pump might explain the differences in the MICs of ciprofloxacin in the isolates.

The significant reduction in ciprofloxacin MICs in the presence of PAβN indicated that overexpression of the RND efflux pump AcrAB-TolC plays a crucial role in the development of fluoroquinolone resistance in *S. Enteritidis*. Efflux pumps are known to contribute to multidrug resistance by actively expelling antibiotics from bacterial cells [17,18,22]. The ability of PAβN to restore susceptibility to ciprofloxacin in many of the isolates suggested that targeting efflux pumps could be a potential strategy for combatting fluoroquinolone resistance. However, the effectiveness of such a strategy would depend on the specific efflux pump involved and the overall resistance profile of the isolate. Further research is needed to understand the regulation and expression of efflux pumps in *S. Enteritidis* and to develop more effective inhibitors.

### 3.5. The Analysis of Plasmid Transferability

**Figure 3:**
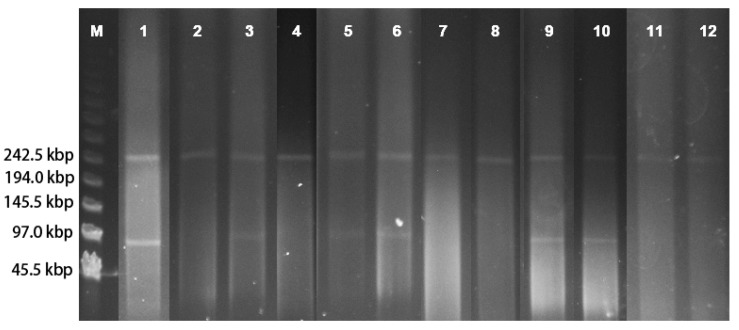
Plasmid profiles of ciprofloxacin-resistant *S. enteritidis* isolates and corresponding transconjugants determined by S1-PFGE. Lane M, A phage lambda ladder used as a molecular size marker with different bands labeled; lane 1, isolate SJTUF11851; lane 2, isolate SJTUF11851-TC; lane 3, isolate SJTUF12065; lane 4, isolate SJTUF12065-TC; lane 5, isolate SJTUF12074; lane 6, isolate SJTUF12092; lane 7, isolate SJTUF12074-TC; lane 8, isolate SJTUF12092-TC; lane 9, isolate SJTUF12122; lane 10, isolate SJTUF12519; lane 11, isolate SJTUF12122-TC; lane 12, SJTUF12519-TC.

Conjugation experiments were performed to identify the transferability of PMQR genes [*aac(6′)-Ib-cr, qnr*A*, qnr*S and *oqx*AB] in fluoroquinolone- and cephalosporin-co-resistant *S. enteritidis* isolates, and *E. coli* C600 was used as the recipient. As shown in Table 2, the PMQR genes were successfully transferred to the recipient in the given isolates. We successfully obtained seven transconjugants, and all these transconjugants were resistant to cephalosporins, ampicillin, kanamycin, gentamicin, tetracycline, trimethoprim-sulfamethoxazole, and nalidixic acid. The MICs of ciprofloxacin in the transconjugants ranged from 0.03 μg/mL to 0.25 μg/mL. When a single PMQR gene (*oqx*AB) was transferred, the MICs of ciprofloxacin in the transconjugants ranged from 0.03 μg/mL to 0.06 μg/mL. On the other hand, when two or three PMQR genes were transferred, the MICs of ciprofloxacin in the transconjugants ranged from 0.125 μg/mL to 0.25 μg/mL. The banding pattern obtained from S1-PFGE indicated that the plasmids present in the transconjugants were approximately 245 kbp in size (Table 2, Figure 3). PCR-based replicon typing revealed the presence of the IncHI2 replicon type in these seven transconjugants (Table 2). The PMQR genes that co-transferred with *bla*_CTX-M-14/55_ on the ~245 kbp IncHI2 plasmid occurred in these seven donor isolates.

The successful transfer of PMQR genes to *E. coli* C600 via conjugation underscored the potential for horizontal gene transfer to facilitate the spread of antibiotic resistance. The presence of multiple resistance determinants, including PMQR genes and ESBL genes, on the same large IncHI2 plasmid (approximately 245 kbp), suggested that these plasmids were capable of carrying and disseminating a wide range of resistance genes. The IncHI2 plasmids were reported to be associated with the carriage of multiple resistance genes and were widely distributed in *Enterobacteriaceae* [34,42]. The finding that the transfer of multiple PMQR genes resulted in higher ciprofloxacin MICs in the transconjugants compared to the transfer of a single PMQR gene highlighted the synergistic effect of multiple resistance mechanisms. This synergy can lead to higher levels of resistance and may complicate efforts to control the spread of antibiotic resistance.

### 3.6. Complete Sequence of Plasmid p12519A

**Figure 4:**
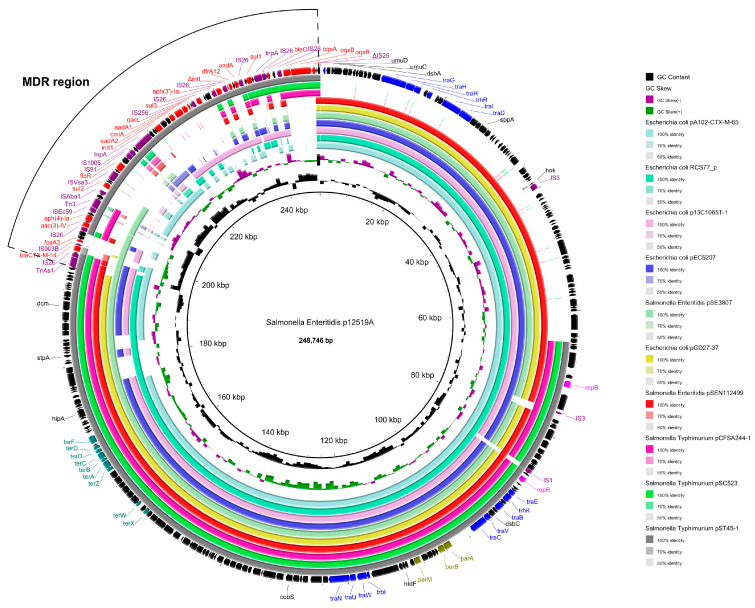
Sequence comparison of *S. enteritidis* plasmid p12519A and other plasmids. Other plasmids are listed as *Escherichia coli* pA102−CTX−M−65 (Accession No. NZ_MN816370), *Escherichia coli* RCS77_p (Accession No. NZ_LT985297), *Escherichia coli* p13C1065T−1 (Accession No. NZ_CP019260), *Escherichia coli* pEC5207 (Accession No. NZ_KT347600), *Escherichia coli* pGD27-37 (Accession No. NZ_MN232191), *S. enteritidis* pSE380T (Accession No. NZ_KY401053), *S. enteritidis* pSEN112499 (Accession No. NZ_KM396299), *S. enteritidis* pCFSA244−1 (Accession No. NZ_CP033253), *S. enteritidis* pST45−1 (Accession No. NZ_CP050754), and *S. enteritidis* pSC523 (Accession No. NZ_KX721511). BLASTN matches with an identity between 50 and 100% are colored in gradient. Boxes or arrows in the outer ring represent the ORFs. Red, antibiotic resistance genes; yellow, IS/transposase; purple, replication-associated genes; teal, heavy metal resistance genes; olive, maintenance/stability genes; black, other genes.

To examine the genomic features of the PMQR-positive plasmid, whole-genome sequencing was conducted on the representative isolate, SJTUF12519, utilizing both the PacBio RS II and Illumina MiSeq systems. The plasmid, designated as p12519A, was identified to be 248,746 base pairs in length and harbored 157 predicted coding sequences (CDSs). Plasmid p12519A harbored IncHI2 (100% identity) and IncHI2A (99.52% identity) replicon types based on the results of PlasmidFinder. A total of 14 antibiotic resistance genes (ARGs) were found in p12519A. Genes responsible for resistance to aminoglycosides [*aac(3)-IV*, *aph(4)-Ia*, *aad*A2, *aad*A1, and *aph(3′)-Ia*], β-lactam (*bla*_CTX-M-14_), fosfomycin (*fos*A3), phenicols (*flo*R and *cml*A), quinolones (*oqx*AB), sulfonamide (*sul*1, *sul*2, and *sul*3), and trimethoprim (*dfr*A12) were identified in plasmid p12519A (Figure 4). In addition, heavy metal resistance genes (*ter*ABCDFWX) were also identified. The conjugational transfer region played a key role in the horizontal spread of IncHI2 plasmids. It was shown in Figure 4 that these IncHI2 plasmids in p12519A contained three conjugational transfer regions of *tra*GHID-*trh*R, *tra*BCEV-*trh*K, and *tra*NUW-*trb*I. The IncHI2 plasmid was known to be transferable and play a key role in the acquisition of antibiotic resistance [43,44]. This plasmid p12519A was similar to IncHI2-type plasmids from *Escherichia coli* and *S. enteritidis* such as *Escherichia coli* pA102-CTX-M-65, RCS77_p, p13C1065T-1, pEC5207, and pGD27-37 as well as *S. enteritidis* pSE380T and pSEN112499 (Figure 4). These plasmids possess a similar backbone but different accessory regions. Accessory regions, which comprised mobile elements (integrons and insertion sequences) and antibiotic resistance genes, were integrated into the conserved plasmid backbone at several sites (Figure 4). Besides the accessory regions, there was a huge difference in the plasmid backbone among p12519A, pCFSA244-1, pST45-1, and pSC523. All three plasmids (pCFSA244-1, pST45-1, and pSC523) were recovered from *S. enteritidis* and lacked a 65,057 bp backbone containing conjugational transfer regions of *tra*G, *tra*H, *tra*I, *tra*D, and *trh*R compared to p12519A. We also observed that IS*1* and IS*3* inserted into sites closed by replicon regions in plasmid p12519A. These findings suggested that IncHI2 plasmids from *S. enteritidis* might have integrated backbone regions from other Gram-negative bacteria such as *Escherichia coli* with the help of mobile genetic elements of ISs and transposons.

In this study, the multidrug resistance region (MRR) likely evolved through the recombination and integration of a variety of ARGs from the local setting with the help of mobile genetic elements, such as ISs and transposons. It is shown in Figure 4 that plasmid p12519A possessed approximately 53,104 bp MRRs. This MRR was a mosaic structure bound at both ends by fragments of IS*26*, and it comprised *bla*_CTX-M-14_, *fos*A3, *oqx*AB, and *cml*A interspersed with different ISs and transposons including IS*26*, IS*Ecp59*, IS*903B*, IS*1006*, IS*Vsa3*, and Tn*3*. The 53.1 kbp MRR of the p12519A was similar to the MRR of *S.* Typhimurium IncHI2 plasmid pST45-1 (Accession No. NZ_CP050754). Both *S. enteritidis* p12519A and *S.* Typhimurium pST45-1 were recovered from diarrheal patients in Shanghai, China (Figure 4).

**Figure 5:**
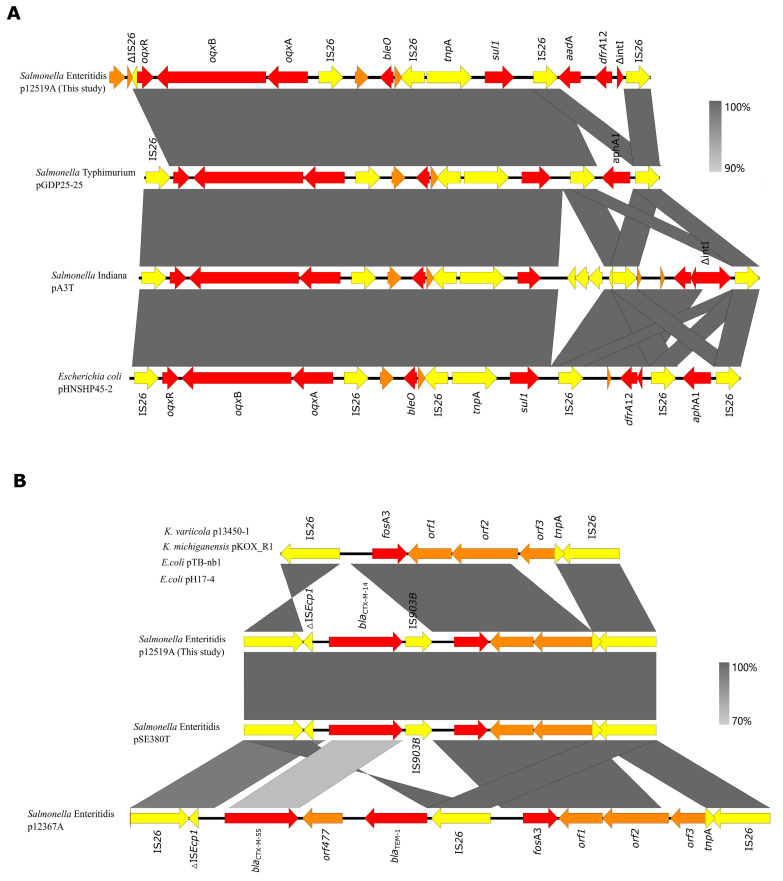
Genetic environment of *oqx*AB (**A**) and *bla*_CTX-M-14_ (**B**) in plasmid p12519A and other plasmids. The plasmids in (**A**) are listed as *S. enteritidis* pGDP25-25 (Accession No. MK673547), *S.* Indiana pA3T (Accession No. KX421096), and *Escherichia coli* pHNSHP45-2 (Accession No. KU341381). The plasmids in (**B**) are listed as *K. variicola* p13450-1 (Accession No. CP026014.1), *K. michiganensis* pKOX_R1 (Accession No. 018107.1), *E. coli* pTB-nb1 (Accession No. CP033632.1), *E. coli* pH17-4 (Accession No. CP021197.1), *S. enteritidis* pSE380T (Accession No. NZ_KY401053), and *S. enteritidis* p12367A (Accession No. NZ_CP041177). Areas shaded in gray indicate homologies between the corresponding genetic loci on each plasmid. Boxes or arrows represent the ORFs. Red, antibiotic resistance genes; yellow, IS/transposase; brown, other genes.

Both ends of *oqx*AB were IS*26*, and this typical transposable structure (IS*26*-*oqx*R-*oqx*AB-IS*26*) was observed in *S. enteritidis* pGDP25-25 (Accession No. MK673547), *S.* Indiana pA3T (Accession No. KX421096), and *Escherichia coli* pHNSHP45-2 (Accession No. KU341381) (Figure 5A). However, truncated IS*26* was observed downstream of *oqx*AB in p12519A (Figure 5A). ΔIS*26* might be truncated by a gene encoding ATP-binding protein downstream. IS*26* plays a pivotal role in the dissemination of ARGs and the fusion of plasmids in bacteria [45,46,47,48,49,50]. However, *fos*A3 was identified in p12519A, which was absent in pST45-1. A *fos*A3 arrangement module (IS*26*-*orf3*-*orf2*-*orf1*-*fos*A3-IS*26*) was observed in *K. variicola* p13450-1 (Accession No. CP026014.1), *K. michiganensis* pKOX_R1 (Accession No. 018107.1), *E. coli* pTB-nb1 (Accession No. CP033632.1), *E. coli* pH17-4 (Accession No. CP021197.1), and *S. enteritidis* p12367A (Accession No. NZ_CP041177) (Figure 5B). The mobilization of the *fos*A3 module carried by the plasmid was likely facilitated by IS*26*.A typical *bla*_CTX-M-14_ transposable structure (IS*26*-ΔIS*Ecp1*-*bla*_CTX-M-14_-IS*903B*) was identified upstream of the *fos*A3 transposition unit (Figure 5B), which was also observed in *S. enteritidis* pSE380T. A similar structure was found in p12367A with *bla*_CTX-M-55_ rather than *bla*_CTX-M-14_, and downstream of *bla*_CTX-M-55_ was *bla*_TEM_-IS*26* rather than IS*903B*. ΔIS*Ecp1*, which might be truncated by IS*26*, has been found to be the most prevalent insertion sequence linked to *bla*_CTX-M_ elements [51]. IS*26* has the ability to mobilize adjacent DNA segments through intramolecular replicative transposition and construct a novel composite transposon via intermolecular replicative transposition. In the absence of RecA-dependent homologous recombination, the Tnp26 transposase facilitates the formation of cointegrates through a conservative reaction between two pre-existing IS*26* elements, which is favored over replicative transposition to a novel location [46]. The incorporation of ARGs into a virulent plasmid was previously identified in *S. enteritidis* , and the fusion process was mediated by IS*26* [50]. Therefore, genomic rearrangements and genetic exchanges were frequently conferred by mobile elements such as plasmids, IS elements, transposons, and integrons, which provided the driving force behind bacterial evolution.

## 4. Conclusions

In conclusion, our study emphasized the emergence of ciprofloxacin resistance in *S. Enteritidis* from patients, attributed to the combined effects of GyrA mutation, PMQR genes, and efflux pump overexpression. The clonal spread and horizontal transfer of isolates accompanying IncHI2 plasmids contributed to the dissemination of PMQR genes including *qnr*, *oqx*AB, and *aac*(6’)-Ib-cr in *S. enteritidis* interspecies. Our results further suggest that the coexistence of *oqx*AB and *bla*_CTX-M-55_ genes on a single IncHI2 plasmid may arise from genomic rearrangements and genetic exchanges facilitated by mobile elements, including ISs, transposons, and integrons. To sum up, our findings underscore the significance of continuous monitoring for the incidence of fluoroquinolone resistance in *S. Enteritidis*, aiding in a deeper understanding of the potential risk it poses to food safety and public health.

## Figures and Tables

**Table 1:** MICs of ciprofloxacin in *S. enteritidis* after treatment with RND efflux pump inhibitor PAβN.

Isolate	Ciprofloxacin		PMQR Genes	GyrA Mutation
	MIC	MIC in the Presence of PaβN ^a^		
SJTUF11851	1	0.125	*aac(6′)-Ib-cr, qnr*A	D87Y
SJTUF12065	1	0.06	*oqx*AB	S83Y-D87Y
SJTUF12074	1	0.06	*oqx*AB	D87Y
SJTUF12092	4	0.5	*aac(6′)-Ib-cr*, *oqx*AB	D87Y
SJTUF12113	2	0.025	*aac(6′)-Ib-cr, qnr*S	D87Y
SJTUF12122	2	0.025	*aac(6′)-Ib-cr, qnr*A*,qnr*S	S83Y
SJTUF12519	1	0.06	*oqx*AB	D87Y

^a^ Isolates with treatment of 0.25-fold MIC of PAβN.

**Table 2:** Characteristics of *S. enteritidis* isolates with co-resistance to ciprofloxacin and ceftriaxone and their transconjugants.

Isolate ^a^	MIC (µg/mL)		Other Resistant Profiles	PMQR Genes	ESBL Gene	Replicon Types ^b^	Plasmid Sizes (kb)
	CRO	CIP					
SJTUF11851	≥128	1	AMP-TIO-CAZ-CTX-KAN-GEN-TET-SXT-NAL	*aac(6′)-Ib-cr, qnr*A	CTX-M-55	IncHI2, NT	~245, ~80
SJTUF11851-TC	64	0.25	AMP-TIO-CAZ-CTX-KAN-GEN-TET-SXT	*aac(6′)-Ib-cr, qnr*A	CTX-M-55	IncHI2	~245
SJTUF12065	≥128	1	AMP-TIO-CAZ-CTX-KAN-GEN-TET-SXT-NAL	*oqx*AB	CTX-M-55	IncHI2, NT	~245, ~80
SJTUF12065-TC	64	0.06	AMP-TIO-CAZ-CTX-KAN-GEN-TET-SXT	*oqx*AB	CTX-M-55	IncHI2	~245
SJTUF12074	≥128	1	AMP-TIO-CAZ-CTX-FOX-KAN-GEN-TET-SXT-NAL	*oqx*AB	CTX-M-55	IncHI2, NT	~245, ~80
SJTUF12074-TC	64	0.03	AMP-TIO-CAZ-CTX-FOX-KAN-GEN-TET-SXT	*oqx*AB	CTX-M-55	IncHI2	~245
SJTUF12092	≥128	4	AMP-TIO-CAZ-CTX-FOX-KAN-GEN-TET-SXT-NAL	*aac(6′)-Ib-cr*, *oqx*AB	CTX-M-55	IncHI2, NT	~245, ~80
SJTUF12092-TC	64	0.125	AMP-TIO-CAZ-CTX-KAN-GEN-TET-SXT	*aac(6′)-Ib-cr,oqx*AB	CTX-M-55	IncHI2	~245
SJTUF12113	≥128	2	AMP-TIO-CAZ-CTX-KAN-GEN-TET-SXT-NAL	*aac(6′)-Ib-cr, qnr*S	CTX-M-55	IncHI2, NT	~245, ~80
SJTUF12113-TC	64	0.25	AMP-TIO-CAZ-CTX-KAN-GEN-TET-SXT	*aac(6′)-Ib-cr, qnr*S	CTX-M-55	IncHI2	~245
SJTUF12122	≥128	2	AMP-TIO-CAZ-CTX-KAN-GEN-TET-SXT-NAL	*aac(6′)-Ib-cr, qnr*A*,qnr*S	CTX-M-14	IncHI2, NT	~245, ~80
SJTUF12122-TC	64	0.25	AMP-TIO-CAZ-CTX-KAN-GEN-TET-SXT	*aac(6′)-Ib-cr, qnr*A*,qnr*S	CTX-M-14	IncHI2	~245
SJTUF12519	≥128	1	AMP-TIO-CAZ-CTX-KAN-GEN-TET-SXT-NAL	*oqx*AB	CTX-M-14	IncHI2, NT	~245, ~80
SJTUF12519-TC	64	0.03	AMP-TIO-CAZ-CTX-KAN-GEN-TET-SXT	*oqx*AB	CTX-M-14	IncHI2	~245

^a^ TC, transconjugant. ^b^ NT, the plasmid replicon was nontypeable.

## Data Availability

The original contributions presented in the study are included in the article/Supplementary Materials, further inquiries can be directed to the corresponding author.

## Supplementary Materials

The following supporting information can be downloaded at: https://www.mdpi.com/article/10.3390/foods14020289/s1, Table S1: Primers used for detection and sequencing of target genes; The detailed information regarding 34 strains of *Salmonella enteritidis* that exhibit resistance to ciprofloxacin is available in an Excel format. Refs. [52,53,54] are cited in Supplementary Materials.

## References

[1] Ferrari R.G., Rosario D.K.A., Cunha-Neto A., Mano S.B., Figueiredo E.E.S., Conte-Junior C.A. (2019). Worldwide Epidemiology of *Salmonella* Serovars in Animal-Based Foods: A Meta-Analysis. Appl. Environ. Microbiol..

[2] Ma Y., Li M., Xu X., Fu Y., Xiong Z., Zhang L., Qu X., Zhang H., Wei Y., Zhan Z. (2018). High-Levels of Resistance to Quinolone and Cephalosporin Antibiotics in MDR-ACSSuT *Salmonella enterica* Serovar Enteritidis Mainly Isolated from Patients and Foods in Shanghai, China. Int. J. Food Microbiol..

[3] Sher A.A., Mustafa B.E., Grady S.C., Gardiner J.C., Saeed A.M. (2021). Outbreaks of Foodborne *Salmonella enteritidis* in the United States between 1990 and 2015: An Analysis of Epidemiological and Spatial-Temporal Trends. Int. J. Infect. Dis..

[4] (2018). The European Union Summary Report on Trends and Sources of Zoonoses, Zoonotic Agents and Food-Borne Outbreaks in 2017. EFSA J..

[5] Monte D.F.M., Sellera F.P. (2020). Salmonella. Emerg. Infect. Dis..

[6] Hu Y., He Y., Wang Y., Fanning S., Cui S., Chen Q., Liu G., Chen Q., Zhou G., Yang B. (2017). Serovar Diversity and Antimicrobial Resistance of Non-Typhoidal *Salmonella enterica* Recovered from Retail Chicken Carcasses for Sale in Different Regions of China. Food Control.

[7] Li Y., Pei X., Zhang X., Wu L., Liu Y., Zhou H., Ma G., Chen Q., Liang H., Yang D. (2019). A Surveillance of Microbiological Contamination on Raw Poultry Meat at Retail Markets in China. Food Control.

[8] Yang X., Huang J., Zhang Y., Liu S., Chen L., Xiao C., Zeng H., Wei X., Gu Q., Li Y. (2020). Prevalence, Abundance, Serovars and Antimicrobial Resistance of *Salmonella* Isolated from Retail Raw Poultry Meat in China. Sci. Total Environ..

[9] Yu X., Zhu H., Bo Y., Li Y., Zhang Y., Liu Y., Zhang J., Jiang L., Chen G., Zhang X. (2021). Prevalence and Antimicrobial Resistance of *Salmonella enterica* Subspecies Enterica Serovar Enteritidis Isolated from Broiler Chickens in Shandong Province, China, 2013–2018. Poult. Sci..

[10] Hald T., Aspinall W., Devleesschauwer B., Cooke R., Corrigan T., Havelaar A.H., Gibb H.J., Torgerson P.R., Kirk M.D., Angulo F.J. (2016). World Health Organization Estimates of the Relative Contributions of Food to the Burden of Disease Due to Selected Foodborne Hazards: A Structured Expert Elicitation. PLoS ONE.

[11] Harris P.N.A., Tambyah P.A., Paterson D.L. (2015). Beta-Lactam and Beta-Lactamase Inhibitor Combinations in the Treatment of Extended-Spectrum Beta-Lactamase Producing Enterobacteriaceae: Time for a Reappraisal in the Era of Few Antibiotic Options?. Lancet Infect. Dis..

[12] Liu H.H. (2010). Safety Profile of the Fluoroquinolones Focus on Levofloxacin. Drug Saf..

[13] Chen K., Yang C., Dong N., Xie M., Ye L., Chan E.W.C., Chen S. (2020). Evolution of Ciprofloxacin Resistance-Encoding Genetic Elements in *Salmonella*. mSystems.

[14] Kuang D., Zhang J., Xu X., Shi W., Chen S., Yang X., Su X., Shi X., Meng J. (2018). Emerging High-Level Ciprofloxacin Resistance and Molecular Basis of Resistance in *Salmonella enterica* from Humans, Food and Animals. Int. J. Food Microbiol..

[15] Wei X., You L., Wang D., Huang H., Li S., Wang D. (2019). Antimicrobial Resistance and Molecular Genotyping of *Salmonella enterica* Serovar Enteritidis Clinical Isolates from Guizhou Province of Southwestern China. PLoS ONE.

[16] Hooper D.C., Jacoby G.A. (2015). Mechanisms of Drug Resistance: Quinolone Resistance: Mechanisms of Quinolone Resistance. Ann. N. Y. Acad. Sci..

[17] Chang M.-X., Zhang J.-F., Sun Y.-H., Li R.-S., Lin X.-L., Yang L., Webber M.A., Jiang H.-X. (2021). Contribution of Different Mechanisms to Ciprofloxacin Resistance in *Salmonella* Spp.. Front. Microbiol..

[18] Zhang Z., Yang J., Xu X., Zhou X., Shi C., Zhao X., Liu Y., Shi X. (2020). Co-Existence of *mph*A, *oqx*AB and *bla*_CTX-M-65_ on the IncHI2 Plasmid in Highly Drug-Resistant *Salmonella enterica* Serovar Indiana ST17 Isolated from Retail Foods and Humans in China. Food Control.

[19] Zhang Z., Meng X., Wang Y., Xia X., Wang X., Xi M., Meng J., Shi X., Wang D., Yang B. (2014). Presence of *qnr*, *aac(6’)-Ib*, *qep*A, *oqx*AB, and Mutations in Gyrase and Topoisomerase in Nalidixic Acid-Resistant *Salmonella* Isolates Recovered from Retail Chicken Carcasses. Foodborne Pathog. Dis..

[20] Li Y., Yang X., Zhang J., Yang S., Zhang S., Chen M., Xue L., Ding Y., Zeng H., Gu Q. (2021). Molecular Characterisation of Antimicrobial Resistance Determinants and Class 1 Integrons of *Salmonella enterica* Subsp. Enterica Serotype Enteritidis Strains from Retail Food in China. Food Control.

[21] Nolivos S., Cayron J., Dedieu A., Page A., Delolme F., Lesterlin C. (2019). Role of AcrAB-TolC Multidrug Efflux Pump in Drug-Resistance Acquisition by Plasmid Transfer. Science.

[22] Li L., Liao X.-P., Liu Z.-Z., Huang T., Li X., Sun J., Liu B.-T., Zhang Q., Liu Y.-H. (2014). Co-Spread of *oqx*AB and *bla*_CTX-M-9G_ in Non-Typhi *Salmonella enterica* Isolates Mediated by ST2-IncHI2 Plasmids. Int. J. Antimicrob. Agents.

[23] Lin D., Chen K., Wai-Chi Chan E., Chen S. (2015). Increasing Prevalence of Ciprofloxacin-Resistant Food-Borne *Salmonella* Strains Harboring Multiple PMQR Elements but Not Target Gene Mutations. Sci. Rep..

[24] Soares F.B., Camargo C.H., Cunha M.P.V., de Almeida E.A., de Jesus Bertani A.M., de Carvalho E., de Paiva J.B., Fernandes S.A., Tiba-Casas M.R. (2019). Co-Occurrence of *qnr*E1 and *bla*_CTX-M-8_ in IncM1 Transferable Plasmids Contributing to MDR in Different *Salmonella* Serotypes. J. Antimicrob. Chemother..

[25] Chen K., Dong N., Chan E.W.-C., Chen S. (2019). Transmission of Ciprofloxacin Resistance in *Salmonella* Mediated by a Novel Type of Conjugative Helper Plasmids. Emerg. Microbes Infect..

[26] Chen K., Dong N., Zhao S., Liu L., Li R., Xie M., Lin D., Wai-Chi Chan E., Meng J., McDermott P.F. (2018). Identification and Characterization of Conjugative Plasmids That Encode Ciprofloxacin Resistance in *Salmonella*. Antimicrob. Agents Chemother..

[27] Thiyagarajan Y., Harish B. (2016). Prevalence of Plasmid-Mediated Quinolone Resistance Genes among Ciprofloxacin-Resistant Clinical Isolates of Enterobacteriaceae over Four Years: A Descriptive Study. Int. J. Infect. Dis..

[28] Chen K., Chan E.W.C., Chen S. (2019). Evolution and Transmission of a Conjugative Plasmid Encoding Both Ciprofloxacin and Ceftriaxone Resistance in *Salmonella*. Emerg. Microbes Infect..

[29] CLSI (Clinical and Laboratory Standards Institute) (2019). Performance Standards for Antimicrobial Susceptibility Testing.

[30] ECAST (2019). The European Committee on Antimicrobial Susceptibility Testing. Breakpoint Tables for Interpretation of MICs and Zone Diameters. Version 9.0. https://www.eucast.org/clinical_breakpoints.

[31] Qiao J., Zhang Q., Alali W.Q., Wang J., Meng L., Xiao Y., Yang H., Chen S., Cui S., Yang B. (2017). Characterization of Extended-Spectrum β-Lactamases (ESBLs)-Producing *Salmonella* in Retail Raw Chicken Carcasses. Int. J. Food Microbiol..

[32] Xu L. (2005). Rapid and Simple Detection of blaCTX-M Genes by Multiplex PCR Assay. J. Med. Microbiol..

[33] Zhang Z., He S., Xu X., Chang J., Zhan Z., Cui Y., Shi C., Shi X. (2022). Antimicrobial Susceptibility and Molecular Characterization of *Salmonella enterica* Serovar Indiana from Foods, Patients, and Environments in China during 2007–2016. Food Control.

[34] Zhang Z., Hu M., Xu X., Lv C., Shi C. (2024). Dynamic Antimicrobial Resistance and Phylogenomic Structure of *Salmonella* Typhimurium from 2007 to 2019 in Shanghai, China. Microbiol. Spectr..

[35] Carattoli A., Bertini A., Villa L., Falbo V., Hopkins K.L., Threlfall E.J. (2005). Identification of Plasmids by PCR-Based Replicon Typing. J. Microbiol. Methods.

[36] Kehrenberg C., de Jong A., Friederichs S., Cloeckaert A., Schwarz S. (2007). Molecular Mechanisms of Decreased Susceptibility to Fluoroquinolones in Avian *Salmonella* Serovars and Their Mutants Selected during the Determination of Mutant Prevention Concentrations. J. Antimicrob. Chemother..

[37] Koren S., Walenz B.P., Berlin K., Miller J.R., Bergman N.H., Phillippy A.M. (2017). Canu: Scalable and Accurate Long-Read Assembly via Adaptive k-Mer Weighting and Repeat Separation. Genome Res..

[38] Bankevich A., Nurk S., Antipov D., Gurevich A.A., Dvorkin M., Kulikov A.S., Lesin V.M., Nikolenko S.I., Pham S., Prjibelski A.D. (2012). SPAdes: A New Genome Assembly Algorithm and Its Applications to Single-Cell Sequencing. J. Comput. Biol..

[39] Walker B.J., Abeel T., Shea T., Priest M., Abouelliel A., Sakthikumar S., Cuomo C.A., Zeng Q., Wortman J., Young S.K. (2014). Pilon: An Integrated Tool for Comprehensive Microbial Variant Detection and Genome Assembly Improvement. PLoS ONE.

[40] Baucheron S., Imberechts H., Chaslus-Dancla E., Cloeckaert A. (2002). The AcrB Multidrug Transporter Plays a Major Role in High-Level Fluoroquinolone Resistance in *Salmonella enterica* Serovar Typhimurium Phage Type DT204. Microb. Drug Resist..

[41] Zhao H., Chen W., Xu X., Zhou X., Shi C. (2018). Transmissible ST3-IncHI2 Plasmids Are Predominant Carriers of Diverse Complex IS*26*-Class 1 Integron Arrangements in Multidrug-Resistant *Salmonella*. Front. Microbiol..

[42] Haenni M., Metayer V., Jarry R., Drapeau A., Puech M.-P., Madec J.-Y., Keck N. (2020). Wide Spread of *bla*_CTX-M-9_/*mcr-9* IncHI2/ST1 Plasmids and CTX-M-9-Producing Escherichia Coli and Enterobacter Cloacae in Rescued Wild Animals. Front. Microbiol..

[43] Shang D., Zhao H., Xu X., Arunachalam K., Chang J., Bai L., Shi C. (2021). Conjugative IncHI2 Plasmid Harboring Novel Class 1 Integron Mediated Dissemination of Multidrug Resistance Genes in *Salmonella* Typhimurium. Food Control.

[44] Du P., Liu D., Song H., Zhang P., Li R., Fu Y., Liu X., Jia J., Li X., Fanning S. (2020). Novel IS26-Mediated Hybrid Plasmid Harboring Tet(X4) in Escherichia Coli. J. Glob. Antimicrob. Resist..

[45] Harmer C.J., Hall R.M. (2015). IS*26*-Mediated Precise Excision of the IS*26*-aphA1a Translocatable Unit. mBio.

[46] Harmer C.J., Hall R.M. (2016). IS*26*-Mediated Formation of Transposons Carrying Antibiotic Resistance Genes. mSphere.

[47] Oliva M., Monno R., Addabbo P., Pesole G., Scrascia M., Calia C., Dionisi A.M., Chiara M., Horner D.S., Manzari C. (2018). IS*26* Mediated Antimicrobial Resistance Gene Shuffling from the Chromosome to a Mosaic Conjugative FII Plasmid. Plasmid.

[48] Partridge S.R., Zong Z., Iredell J.R. (2011). Recombination in IS*26* and Tn*2* in the Evolution of Multiresistance Regions Carrying *bla*_CTX-M-15_ on Conjugative IncF Plasmids from Escherichia Coli. Antimicrob. Agents Chemother..

[49] Wong M.H.-Y., Chan E.W.-C., Chen S. (2017). IS*26*-Mediated Formation of a Virulence and Resistance Plasmid in *Salmonella enteritidis*. J. Antimicrob. Chemother..

[50] Li C., Zhang Z., Xu X., He S., Zhao X., Cui Y., Zhou X., Shi C., Liu Y., Zhou M. (2021). Molecular Characterization of Cephalosporin-Resistant *Salmonella enteritidis* ST11 Isolates Carrying *bla*_CTX-M_ from Children with Diarrhea. Foodborne Pathog. Dis..

[51] Cattoir V., Poirel L., Rotimi V., Soussy C.-J., Nordmann P. (2007). Multiplex PCR for Detection of Plasmid-Mediated Quinolone Resistance Qnr Genes in ESBL-Producing Enterobacterial Isolates. J. Antimicrob. Chemother..

[52] Chen X., Zhang W., Pan W., Yin J., Pan Z., Gao S., Jiao X. (2012). Prevalence of qnr, aac(6’)-Ib-cr, qepA, and oqxAB in Escherichia coli isolates from humans, animals, and the environment. Antimicrob. Agents Chemother..

[53] Eaves D.J., Randall L., Gray D.T., Buckley A., Woodward M.J., White A.P., Piddock L.J.V. (2004). Prevalence of mutations within the quinolone resistance-determining region of gyrA, gyrB, parC, and parE and association with antibiotic resistance in quinolone-resistant Salmonella enterica. Antimicrob. Agents Chemother..

[54] Park C.H., Robicsek A., Jacoby G.A., Sahm D., Hooper D.C. (2006). Prevalence in the United States of aac(6’)-Ib-cr encoding a ciprofloxacin-modifying enzyme. Antimicrob. Agents Chemother..

