# Supplementary material for: Clonal Spread and Genetic Mechanisms Underpinning Ciprofloxacin Resistance in Salmonella enteritidis
Source: Foods. 2025 Jan 16;14(2):289. doi: 10.3390/foods14020289 (PMC11765419; doi:10.3390/foods14020289)
Supplement: Supplementary file 1 [file foods-14-00289-s001.zip › foods-3387332-supplementary/Supplementary materials/Table S1.pdf]

Supplementary Data

Table S1 Primers used for detection and sequencing of target genes

Target genes	Primers	Sequence (5′-3′)	Annealing temperature (℃)	Product size(bp)	Reference
QRDR gene					
*gyr*A	*gyr*A-F<br>*gyr*A-R	ACGTACTAGGCAATGACTGG<br>AGAAGTCGCCGTCGATAGAA	56	190	(Eaves et al., 2004)
*gyr*B	*gyr*B-F<br>*gyr*B-R	GCGCTGTCCGAACTGTACCT<br>GATCAGCGTCGCCACTTCC	63	181	(Eaves et al., 2004)
*par*C	*par*C-F<br>*par*C-R	CTATGCGATGTCAGAGCTGG<br>TAACAGCAGCTCGGCGTATT	62	270	(Eaves et al., 2004)
*par*E	*par*E-F<br>*par*E-R	TCTCTTCCGATGAAGTGCTG<br>ATACGGTATAGCGGCGGTAG	64	240	(Eaves et al., 2004)
PMQR gene					
*qnr*A	*qnr*A-F<br>*qnr*A-R	AGAGGATTTCTCACGCCAGG<br>TGCCAGGCACAGATCTTGAC	60	580	(Cattoir et al., 2007)
*qnr*B	*qnr*B-F<br>*qnr*B-R	GGMATHGAAATTCGCCACTG<br>TTTGCYGYYCGCCAGTCGAA	56	264	(Cattoir et al., 2007))
*qnr*S	*qnr*S-F<br>*qnr*S-R	GCAAGTTCATTGAACAGGGT<br>TCTAAACCGTCGAGTTCGGCG	57	428	(Cattoir et al., 2007)
*qep*A	*qep*A-F<br>*qep*A-R	CTGCAGGTACTGCGTCATG<br>CGTGTTGCTGGAGTTCTTC	60	403	(Chen et al., 2012b)
*oqx*A	*oqx*A-F<br>*oqx*A-R	GACAGCGTCGCACAGAATG<br>GGAGACGAGGTTGGTATGGA	62	339	(Chen et al., 2012b)
*oqx*B	*oqx*B-F<br>*oqx*B-R	CGAAGAAAGACCTCCCTACCC<br>CGCCGCCAATGAGATACA	62	240	(Chen et al., 2012b)
aac(6')-Ib	*aac(6')-Ib*-F<br>*aac(6')-Ib*-R	TTGCGATGCTCTATGAGTGGCTA<br>CTCGAATGCCTGGCGTGTTT	55	482	(Park et al., 2006)

Reference

Cattoir, V., Poirel, L., Rotimi, V., Soussy, C.-J., Nordmann, P., 2007. Multiplex PCR for detection of plasmid-mediated quinolone resistance qnr genes in ESBL-producing enterobacterial isolates. J. Antimicrob. Chemother. 60, 394–397. https://doi.org/10.1093/jac/dkm204

Chen, X., Zhang, W., Pan, W., Yin, J., Pan, Z., Gao, S., Jiao, X., 2012. Prevalence of qnr, aac(6’)-Ib-cr, qepA, and oqxAB in Escherichia coli isolates from humans, animals, and the environment. Antimicrob. Agents Chemother. 56, 3423–3427. https://doi.org/10.1128/AAC.06191-11

Eaves, D.J., Randall, L., Gray, D.T., Buckley, A., Woodward, M.J., White, A.P., Piddock, L.J.V., 2004. Prevalence of mutations within the quinolone resistance-determining region of gyrA, gyrB, parC, and parE and association with antibiotic resistance in quinolone-resistant Salmonella enterica. Antimicrob. Agents Chemother. 48, 4012–4015. https://doi.org/10.1128/AAC.48.10.4012-4015.2004

Park, C.H., Robicsek, A., Jacoby, G.A., Sahm, D., Hooper, D.C., 2006. Prevalence in the United States of aac(6 ’)-Ib-cr encoding a ciprofloxacin-modifying enzyme. Antimicrob. Agents Chemother. 50, 3953–3955. https://doi.org/10.1128/AAC.00915-06
